# Neurorehabilitation needs a qualitative perspective: a case exemplar from stroke recovery and rehabilitation

**DOI:** 10.3389/fnhum.2026.1707789

**Published:** 2026-02-26

**Authors:** Marika Demers, Carolee J. Winstein

**Affiliations:** 1 Institut Universitaire sur la Réadaptation en Déficience Physique de Montréal, Center for Interdisciplinary Research in Rehabilitation of Greater Montreal, CIUSSS du Centre-Sud-de-l’Île-de-Montréal, Montreal, QC, Canada; 2 Faculty of Medicine, School of Rehabilitation, Université de Montréal, Montreal, QC, Canada; 3 Division of Biokinesiology and Physical Therapy, Herman Ostrow School of Dentistry, and Department of Neurology, Keck School of Medicine, University of Southern California, Los Angeles, CA, United States

**Keywords:** stroke, neurosciences, rehabilitation, mixed methods, recovery

## Abstract

Neurorehabilitation is a medical process using neuroplasticity to help people recover from nervous system damage (like stroke, injury or disease) by improving function, independence, and quality of life through therapies (physio, occupational, speech) to retrain the brain and learn new ways to perform daily tasks, addressing physical, cognitive, and emotional needs. This process is shaped by the dynamic interaction of the person, the environment, and the task. Quantitative methods rooted in the science of experience-dependent plasticity, and rigorous clinical trial designs have produced significant advances, including the development of novel neurotechnologies. However, the comprehensive translation of these advances into meaningful outcomes for people living with a neurological condition requires a broader perspective. Central to this perspective is the recognition that the recovery process, includes motor/physical, sensory/perceptual, cognitive, affective, and psychosocial dimensions. We emphasize the integration of qualitative methods into neurorehabilitation research to provide for better translation and a more comprehensive understanding of the process. This perspective is organized into four thematic sections: foundations/current issues; integration of lived experience to improve research and current practice; recommendations for behavioral interventions; and integration of qualitative methods into clinically less mature topics to reveal mechanisms that quantitative data alone cannot capture.

## 1 Foundations and current issues

The field of neurorehabilitation, involves the interaction of the person, the environment, and the task (ScienceDirect, 2026). Although neurorehabilitation spans multiple conditions, we center this perspective on post-stroke recovery and rehabilitation which we believe exemplifies principles that generalize across the field. Stroke is one of the most prevalent neurological medical diagnosis for which a majority of the recovery research has been focused. In an attempt to provide unambiguous definitions of motor recovery and compensation using the framework of the World Health Organization International Classification of Functioning (ICF), Levin et al. (2009) define recovery of motor performance as the reappearance of elemental motor patterns present prior to central nervous system injury. They define motor compensation as the emergence of new motor patterns through adaptation or substitution of remaining motor elements, whereby functions are taken over, replaced or substituted by different end effectors (i.e., hand or foot) or body segments. While Levin et al. (2009) make an important and fundamental distinction between recovery and compensation, their point of view is limited to the motor system or physical domain, where their classification is based on the first two ICF body functions/structures and activity levels. They do not extend the classification to the participation level of the ICF because clear distinctions between processes of recovery and compensation are more difficult to identify. Research in the motor/physical domain demonstrates greater research maturity, evidenced by the substantially larger volume of high-quality RCTs [between 1970 and 2012, 1,063 + trials vs. 64 for cognitive rehabilitation (McIntyre et al., 2020)], enabling more systematic operationalization through consensus on measurement approaches, standardized outcome tools, and robust evidence synthesis. In contrast, cognitive, perceptual, and affective domains, while not necessarily of lower methodological quality in individual studies, lack the critical mass of research needed to achieve comparable rigor and systematization (McIntyre et al., 2020; Bertoni et al., 2024; Kwakkel et al., 2017; White and Lockwood, 2025).

For this perspective, we embrace a more comprehensive approach to understanding recovery and compensation that includes physical/motor, cognitive, sensory/perceptual and affective domains. We argue that integrating qualitative methods with quantitative methods can help explore non-motor processes such as the cognitive, perceptual and emotional needs/impairments that have been less studied but are integral to the rehabilitation process. Increased physical recovery (including adaptation and compensation) does not always lead to improved cognitive, perceptual and emotional status. For example, while approximately 26% of mild-to-moderately impaired stroke survivors remained depressed at the 12-month mark (Dong et al., 2021), a separate 6-year follow-up study found that even mild depression symptoms were seen in patients who primarily experienced mild strokes with mild to no motor impairment (Ytterberg et al., 2022). Thus, even those with mild stroke impairment (i.e., good physical recovery) can and do experience depression symptoms. These non-motor/physical domains are important for garnering the necessary engagement and motivation in the neurorehabilitation process. Further, distinguishing depression from apathy (see below) is important for designing a patient-specific intervention and understanding the unique responsiveness to that intervention. Clinical research pertaining to proper assessment and identification of deficiencies in these complimentary domains (e.g., depression, resilience, apathy) is important not only for the field of neurorehabilitation, but for implementation and the success of the rehabilitation process itself.

Motor/physical outcomes have historically been emphasized in neurorehabilitation trials, while cognitive, sensory/perceptual, and affective outcomes have been comparatively under-measured and under-integrated. In a 2018 topical review, Quinn et al. (2018) recognized that current stroke practice has predominantly prioritized physical manifestations, often overlooking neuropsychological factors. Therefore, we argue that now, to truly advance the field and translate the neuro-behavioral science into meaningful outcomes for the individual person, we need to understand more about the stroke survivor’s perspective in the context of the recovery trajectory. This is not a new idea; it was first proposed in the 2011 McDonnell project (Corbetta and Fitzpatrick, 2011). Our perspective is that what the stroke survivor brings to the rehabilitation process, not merely the level of physical brain damage (e.g., lesion size and location) and movement impairment, but their cognitive, sensory/perceptual and emotional needs and deficits, are critically important for developing patient-specific interventions, and building testable hypotheses that will ultimately benefit the individual stroke survivor.

Current issues that challenge the field are described in the next sub-section. Here we give some examples to expand the challenges inherent in clinical integration, translational pathways, and measurement sensitivity that impact neurorehabilitation research and practice. This perspective builds in part on our prior empirical and conceptual work; however, we integrate these insights with independent literature to situate them within the broader stroke and neurorehabilitation science field.

First, the randomized clinical trial approach, mostly in the motor/physical domain, has had mixed effects on advancing the field, in part because of the tremendous variability between persons in their responsiveness to recovery-promoting interventions. Given that recovery processes are multifaceted and complex, interventions usually have multiple essential ingredients; thus a single primary outcome measure rarely captures the effects of that complex intervention (Winstein et al., 2016; Lewthwaite et al., 2018). Consequently, clinical trials in neurorehabilitation do not easily fit the mold established, for example, with drug or device trials. However, the stages or phases of clinical trial progression are generally similar (e.g., Phase I, II, III, IV) (Campbell et al., 2000; Dobkin, 2009). Part of the problem is the limited foundational knowledge (e.g., observational and/or natural history) to determine a viable mechanism(s) of action (MoA). Often, a generic MoA (e.g., experience-dependent plasticity) is postulated but specific intervention components are rarely mapped to it. When it is done, the comparison group is often described with little detail as the “standard of care.” The problem is that standard care varies considerably across the country, due in part to the disconnect between research and practice. We encourage detailed protocol descriptions of usual care or the comparison group intervention, acknowledging center- and country-level variability, and ensuring each participants’ care is traceable (Hoffmann et al., 2014; Negrini et al., 2020).

The EXCITE trial of constraint-induced movement therapy (CIMT) for stroke survivors was the first successful randomized clinical trial of a neurorehabilitation intervention (Wolf et al., 2006). With 222 stroke survivors (small by comparison for clinical trials of medical interventions in stroke), EXCITE showed that 60 h of one-on-one therapy during the subacute or chronic phase (cross-over delayed group) was better than whatever the control group got (which was often nothing) during a two-week period after acute rehabilitation discharge. Importantly, this trial was testing for efficacy, not effectiveness of the intervention. While this was a landmark study that moved the needle forward in the field of motor rehabilitation (Luft and Hanley, 2006), the clinical practicality of administering the intervention (e.g., therapist guided task practice for 6 h/day for 2 weeks while wearing a constraint mitt on the less-affected side during waking hours) was low and more importantly, we learned very little about the essential MoA from the EXCITE trial. Was it the Mitt? Was it the task practice dosage? Was it the transfer package? (See expanded discussion of the transfer package below). Since increased practice dosage (i.e., task-specific practice) is accepted by the scientific community to benefit motor recovery, funding agencies now expect comparisons with mechanistically-motivated control groups (e.g., dose equivalent; active vs. passive; with/without neurotechnology). This concern stems in part from little progress in specifying the therapeutic ingredients (mentioned above) and processes that cause measured changes in patient functioning. The hypothesized MoA remains undeveloped and insufficiently integrated into the design of viable interventions (Kimberley and Plow, 2025).

Whyte et al. (2020) provide a useful theoretical framework, the rehabilitation treatment specification system (RTSS) (Van Stan et al., 2019). RTSS aims to improve rehabilitation research reporting by enabling testing and refinement of treatment theories. It was developed to address the lack of a system to characterize rehabilitation treatments, challenged by: (1) unclear guidance on which details relate to changes in patient function—essential for research reporting and knowledge development; (2) descriptions focused on therapist type or problem addressed rather than therapy content (e.g., 30 min of occupational therapy); and (3) a lack of a uniform, standard, cross-discipline system for describing treatment (Van Stan et al., 2019). While the RTSS can arguably provide the needed guidance to describe a treatment protocol, improve study replication and evidence synthesis, the uptake in clinical research and practice remains modest. This might be one area where the field of neurorehabilitation could benefit by using the Bradford Hill criteria for causation, originally developed in epidemiology (Nowinski et al., 2022). This could serve to structure mechanistic claims and strengthen the causal plausibility of observed associations in neurorehabilitation trials. Further, inter-individual variability and heterogeneity of stroke/neurorehabilitation populations, and single-case experimental designs (SCED) are appropriate quantitative designs for mechanism testing at the individual level (for a practical guide, see Krasny-Pacini and Evans, 2018).

The second factor that impacts the current climate in neurorehabilitation is a lack of appreciation for the fundamental sciences underlying the field. This includes the multiple learning and memory mechanisms engaged in the rehabilitation process (Schweighofer et al., 2023; Leech et al., 2021). Around the turn of the millennium, a group of clinician scientists recognized that neurorehabilitation may be at a crossroads: one path leads to a mature clinical-behavioral science grounded in psychology and neuroscience; the other continues the status quo, which under economic pressure risks marginalizing the field, worsening outcomes for stroke survivors and reinforcing social policies that leave those with fewer resources to fend for themselves (Corbetta and Fitzpatrick, 2011). This prescient perspective is still relevant today more than a decade later. While there have been advances, particularly in the neuroscience of brain recovery in general and specifically in response to rehabilitation, the true integration of psychology and neuroscience remains slower to advance. This might be due in part to a latent bias, lack of understanding and training in the use of patient-reported outcomes and qualitative methods (Kayes and McPherson, 2010). This is one of those areas that would benefit from interdisciplinary collaborations. A relevant example, the ENIGMA (enhancing neuroimaging genetics through meta-analysis) Consortium is a worldwide collaborative network of over 1,400 scientists across 43 countries, launched in 2009 to analyze brain imaging, genetic, and clinical data. It seeks to identify genetic influences on brain structure and the neural basis of neurological/psychiatric disorders by pooling data from over 100,000 individuals (Thompson et al., 2020).

Finally, a related problem in the field concerns the valid determination of outcome(s) after stroke, important in both clinical practice and in research trials. Since outcome measures are often used to determine the need for follow-up care, the effectiveness/appropriateness of an intervention for a specific patient and to evaluate the effects of therapy, the selection of the appropriate measure(s) can be a challenge (Barak and Duncan, 2006). Over a decade ago, Stewart and Cramer (2013) reported that patient-reported outcomes provided unique insights into motor function after stroke. One untested notion in motor neurorehabilitation is that the path to recovery (if possible) is often difficult and longer (i.e., investment principle) (Fisher and Woll, 1995), while the alternative path using compensation/adaptation is easier, with more immediate results (Tsay and Winstein, 2021). This raises the concern that taking a compensation path, though immediately rewarding, may limit recovery (through some interference or disuse mechanism such as learned nonuse). However, to our knowledge, other than anecdotal, there is little to no evidence to support this hypothesis and further, we are not aware of any studies that have rigorously tested it. Indeed the phenomenon of “learned non-use” has only recently been systematically investigated using a Delphi approach (Hirsch et al., 2021).

## 2 Integration of lived experience to improve research and current practice

Both qualitative and quantitative methods are powerful for neurorehabilitation research, but their aims and the type of questions answered will differ. While quantitative methods typically use a top-down approach that begins with a testable hypothesis, qualitative methods adopt a bottom-up approach when theory is limited or knowledge is scarce, making them inherently exploratory and well suited for hypothesis generation in complex, context-dependent rehabilitation settings. Qualitative methods encompass research approaches that explore experiences, perceptions, behaviors and meanings through non-numerical data such as interviews, focus groups, observations, and open-ended texts (Teherani et al., 2015; Gill et al., 2008). We believe the integration of qualitative methods with quantitative methods are especially relevant for the field of neurorehabilitation to generate hypotheses on underlying mechanisms that potentially influence recovery more generally and sensorimotor recovery specifically.

Only recently, within the last few years, well-designed mixed methods studies have emerged. For example, Lipson-Smith et al. (2023) conducted a mixed-methods multiple-case study at two inpatient rehabilitation facilities in Victoria, Australia, (*n* = 20 at Case 1, *n* = 16 at Case 2) using “walk-through” semi-structured interviews, behavioral mapping, questionnaires, and a retrospective audit to study the physical environment’s role in stroke recovery. In 2024, other researchers used a two-phase sequential explanatory mixed methods design including a feasibility randomized controlled trial (two arm, assessor blinded) followed by focus groups to determine the feasibility of a self-management intervention to improve mobility in the community after stroke (Sahely et al., 2024). Recent investigations from our group and others have begun to probe the qualitative aspects of stroke survivors to better understand the individual characteristics that predict positive recovery outcomes. In qualitative semi-structured interviews, we explored the perceptions of stroke survivors about factors influencing movement behavior and recovery. We identified mindset, motor capacity, the physical and social environment as key determinants that shape engagement in meaningful activities, which in turn support motor recovery (Cain et al., 2024). These findings offer a more comprehensive perspective on the meaningfulness of personal, environmental, and occupational factors influencing movement and motor recovery post-stroke. Similarly, a meta-synthesis of 18 qualitative studies identified that participation in the first year post-stroke is influenced by personal factors, such as perseverance, adaptability, and emotional resilience, social support and positive interactions with their healthcare professionals (Walsh et al., 2015). Taken together, the perspectives of stroke survivors are essential for providing firsthand insights into the many factors that shape their recovery and community reintegration—insights that may be overlooked in traditional clinical outcome assessments. Once we better understand the persons’ perspective, we can better craft and individualize recovery-promoting interventions to engage them in a journey toward recovery in the broad sense. Incorporating stroke survivors’ voices ensures that support systems and services address actual needs to promote more effective, patient-oriented and sustainable outcomes.

In addition to hypotheses generation, qualitative methods can uncover insights not captured by standardized outcome measures, and explain, complement or contrast quantitative findings (Gill et al., 2008; DiCarlo et al., 2025).^1^ Combining qualitative and quantitative methods in a clinical trial, mechanistic cohort study or SCED constitutes a mixed methods approach. Mixed methods research is defined by Johnson et al. (2007) as the type of research which combines elements of qualitative and quantitative research approaches to enhance both depth and breadth of understanding and corroboration. The flexibility and adaptability of mixed methods design make it relevant for neurorehabilitation researchers seeking a comprehensive and nuanced understanding of diverse research questions. Multiple mixed methods designs exist, each tailored to different research purposes and contexts (Table 1 presents a description of three mixed methods designs).

**Table 1:** Overview of the basic mixed methods designs (Creswell, 2022).

Mixed methods design	Description	Purpose	Example	
Exploratory	Exploratory sequential design begins with qualitative data collection and analysis, followed by quantitative data collection and analysis to help interpret the findings	How can the qualitative findings inform quantitative questions or methods?	Developing a patient-centered exercise program	
			Qualitative phase: Conduct focus groups with stroke survivors to explore perceived barriers, motivations, and preferences related to exercise	
			Quantitative phase: Use findings to design a patient-centered exercise intervention and test its feasibility and acceptability through a randomized control trial	
Explanatory	Explanatory sequential design begins with collecting and analyzing quantitative data, followed by qualitative data collection and analysis to help explain the quantitative results	How do the qualitative findings help explain the quantitative results? Or How do the qualitative findings expand on the experimental outcomes?	Conducting a randomized control trial of a complex sensorimotor intervention paired with a neurotechnology (e.g., vagus nerve stimulation (VNS) + Rehab) (Dawson et al., 2021)	
			Quantitative phase: Conduct a randomized controlled trial with both primary and secondary standardized outcome measures (e.g., Fugl-Meyer Assessment, Wolf Motor Function Test and reaching kinematics)	
			Qualitative phase: Interview stroke survivors to understand their experiences, challenges, and perceptions of the benefit of the intervention, shedding light on why some participants improved while others did not	
Convergent	Convergent parallel design involves collecting and analyzing qualitative and quantitative data separately, then comparing or combining the results to interpret them. It involves the discussion of similarities or differences between qualitative and quantitative of findings	Do the quantitative results and the qualitative findings converge?	Comparing sensorimotor recovery in urban and rural rehabilitation settings	
			Quantitative: Collect standardized sensorimotor outcome measures (e.g., gait speed, strength) from urban and rural clinics	Qualitative: Conduct interviews with stroke survivors and clinicians about access to services, therapy quality, and contextual challenges
			Integration: Examine how environmental and social context may help explain quantitative differences in recovery outcomes	

Mixed methods can be leveraged in neurorehabilitation trials, where both measurable (quantifiable) outcomes and lived experiences are critical. The integration of qualitative and quantitative data can enhance understanding of intervention effectiveness, inform intervention design and refinement, uncover mechanisms and contextual factors. It can also capture lived experiences of stroke to gain a deeper understanding of outcomes and quality of life. Mixed methods are also relevant to guide implementation and improve translational efforts. Since neurorehabilitation remains an evolving area of research and practice, mixed methods research is particularly valuable to integrate the measurable outcomes of recovery with the rich, contextual insights of stroke survivors lived experiences—offering a more comprehensive understanding of recovery interventions, individual variability, and the differing pathways to meaningful recovery. For example, Thompson et al. (2024) compared a state-of-the-art motor therapy intervention—high intensity walking (FAST), to a step activity monitoring behavioral intervention (SAM) or a combined intervention (FAST + SAM). They concluded that only individuals with chronic stroke who completed a step activity monitoring behavioral intervention with skilled coaching and goal progression demonstrated improvements in physical activity (steps/day).

## 3 Recommendations for behavioral interventions

Recent studies drawing on psychology, behavioral medicine and economics offer promising solutions to address persisting challenges in neurorehabilitation. One example is ecological momentary assessment and intervention (EMA and EMI, respectively), which originated in behavioral medicine, primarily to understand pain and its varying course over the day. EMA and EMI can collect real-time data about participants’ experiences and behaviors in the natural environment (Stone and Shiffman, 1994) and deliver timely, context-sensitive interventions based on that information (Heron and Smyth, 2010). In stroke rehabilitation, EMA helps reveal how mood varies throughout daily life (Johnson et al., 2009; Lau et al., 2019; Lee et al., 2020) and how early social interactions and support influence long-term depression and everyday functioning (Villain et al., 2017). Previous work using EMA also identified behavioral risk factors linked to depression (Jean et al., 2013; Vansimaeys et al., 2017; Mazure et al., 2014), as well as relevant factors contributing to post-stroke fatigue (de Vries et al., 2023a; de Vries et al., 2023b; Lau et al., 2023). Our group combined EMA with accelerometry to characterize the momentary effect of social-cognitive factors on arm and hand use in chronic stroke survivors (Chen et al., 2021). The results of this work highlighted the role of social context and self-efficacy in shaping how stroke survivors use their paretic arm and hand in unsupervised everyday activities (Chen et al., 2023). While EMI remains relatively new in neurorehabilitation, it demonstrates potential to extend the repeated within-environment prompting methods of EMA by providing personalized interventions in the real-life context, such as reminders, education and tailored recommendations (Bell et al., 2017; Demers and Winstein, 2021). EMA and EMI are promising for translation to understand the complexities of the biopsychosocial factors influencing sensorimotor recovery outside of the supervised clinical environment and enable more precise, and personalized data-driven interventions (Demers and Winstein, 2021).

Similar to the findings from qualitative work and EMA, growing evidence supports the role of mindset and self-efficacy in driving recovery. The OPTIMAL theory of motor learning (optimizing performance through intrinsic motivation and attention for learning) posits that enhanced expectancies and autonomy support—key elements of a growth mindset and self-efficacy—boost motivation and facilitate more effective motor learning (Wulf and Lewthwaite, 2016). By fostering positive beliefs about one’s ability to succeed and providing a sense of control, the theory leverages essential psychological factors and needs to optimize performance and learning outcomes (see also Cain et al., 2024).

Recent locomotor work out of Reisman’s lab (Thompson et al., 2024) mentioned above, demonstrates the benefits of behavioral approaches compared with pure motor approaches. From a neuroscience perspective, the early work of Gauthier et al. (2008) compared stroke survivors who received all the components of CIMT (i.e., task practice, mitt wear, transfer package) with those who received the signature CIMT protocol but without the transfer package. The ‘transfer package’ was designed to maximally transfer therapeutic gains to real-world activities (i.e., lived experience), included daily monitoring of life situation use of the more affected limb and problem-solving with a therapist or coach to overcome perceived barriers to using the limb. The team found that while both groups showed performance improvements, plastic structural brain changes were only seen in participants who received the transfer package. This finding from a single-site, Phase II RCT found that recovery-promoting brain plasticity is harnessed by the behavioral transfer package component of CIMT but not the other motor elements of CIMT previously thought to be essential. Together, these findings from behavioral interventions, including problem-solving strategies, seem to represent the glue that implements the structural neural network changes needed to achieve durable learning and recovery. This work needs replication, but it provides initial evidence about potential mechanisms important for the translational pathway.

Finally, behavioral economics principles can be leveraged to encourage behavior change—an essential yet complex and multifaceted aspect of neurorehabilitation (Séguin et al., 2024). By integrating psychology and economics, behavioral economics explores how people make decisions in real-life situations—often in ways that deviate from traditional rational models (King et al., 2013; Thaler and Sunstein, 2009). Drawing on behavioral economics, evidence supports the effectiveness of loss-framed incentives, nudges, and tailored feedback in promoting healthier behaviors across populations, including boosting physical activity in adults living with obesity and cardiovascular conditions (Chokshi et al., 2018; Halpern et al., 2015; Jenkins et al., 2019; Patel et al., 2017; Patel et al., 2019). As an example in neurorehabilitation, Waddell et al. (2021) have developed the BE Mobile intervention using commercial-grade wearable sensors and a companion app to increase mobility post-stroke. BE Mobile incorporated gamification elements (e.g., loss-framed points and levels) along with social incentives to achieve daily step goals. Participants assigned to the intervention arm improved their mean daily steps compared to participants in a control group (Waddell et al., 2022). Behavioral economics holds promise for advancing neurorehabilitation, but further research is needed to establish best practices for implementing strategies shown to be effective (Studer and Shubert, 2024).

## 4 Integration of qualitative methods into clinically less mature but promising topics

In this section, we propose integration of qualitative methods into four less clinically mature research areas, specifically in stroke. First is the importance of sleep for neurorehabilitation (Brown et al., 2020; Frange et al., 2023). A recent systematic review (Frange et al., 2023) highlighted that sleep facilitates neuroplasticity and underpins motor and cognitive therapy through consolidation of newly learned skills, with time in REM sleep related to the magnitude of offline motor learning. The authors recommend that sleep should be considered in the treatment plan for successfully targeted rehabilitation efforts to optimize cognitive and motor learning. However, sleep disorders after stroke are highly prevalent (affecting 50–70% of stroke survivors) and multifactorial, ranging from obstructive sleep apnea (the most common, affecting over 60% acutely) to insomnia (30–40%), circadian disruptions, and restless leg syndrome underscoring the need for interdisciplinary collaborations, nuanced assessment and intervention strategies, and a deliberate, careful approach to research. Despite extensive neurophysiologic investigation documenting the bidirectional relationship between sleep and stroke, and the impact of sleep disorders on rehabilitation outcomes, patient perspectives on sleep disturbances—including what aspects of sleep disruption matter most to stroke survivors, how sleep problems affect daily functioning and quality of life, and barriers to implementing recommended sleep interventions—remain critically underexplored. A 2025 systematic literature review found a disconnect between data acquired by digital health technologies for sleep and what is meaningful to patients, highlighting the need to examine the elements of sleep disturbance that are important to measure from the patient perspective and experience (Riedmann et al., 2025). This represents a significant gap where qualitative methods could illuminate the lived experience of sleep disruption and inform more patient-centered, acceptable, and effective interventions.

The second topic, brain–computer interfaces have progressed from proof-of-concept to multicenter clinical trials, with recent studies including nearly 300 patients demonstrating efficacy for upper limb rehabilitation (Wang et al., 2024). However, routine clinical deployment still remains limited, with translation hampered by technical complexity, cost, and the need for specialized expertise. While the evidence base has matured beyond individual case exemplars to include multiple randomized controlled trials and meta-analyses, widespread clinical adoption faces practical barriers. This represents an important area for the integration of qualitative methods. While the evidence base remains primarily neurophysiologic in nature (Séguin et al., 2024), with limited understanding of patient experiences, preferences, and the lived reality of BCI use, emerging participatory design studies are beginning to incorporate stroke survivor perspectives into BCI development (Oliveira et al., 2025). These approaches reveal the critical importance of patient-centered design, individualized treatment protocols, and shared decision-making—perspectives that quantitative efficacy trials alone cannot capture.

Third, apathy, mentioned earlier, is developed by more than one-third of stroke survivors yet rarely studied in relation to stroke neurorehabilitation. For apathy after stroke, a recent systematic review found only a pilot RCT (*n* = 13) of repetitive transcranial magnetic stimulation (Sasaki et al., 2017) and an RCT (*n* = 30) of strategy training (Skidmore et al., 2015), along with open-label studies of pharmacological treatments (e.g., Whyte et al., 2008). Apathy is a neuropsychiatric syndrome defined as a loss of motivation that manifests as a reduction in self-driven, goal-directed activity and not attributable to sedation or motor impairment. Further, apathy is a critical and understudied syndrome that can be distinguished from depression, and as such, a potential benefactor from an integrated mixed methods approach. Studies in older adults and various clinical populations demonstrate that apathy is associated with altered effort-based decision-making behavior. It is a common consequence of neurological damage, as is anosognosia—impaired awareness of deficits—that severely impedes rehabilitation engagement, and outcomes across motor, sensory/perceptual, cognitive, and affective domains. Current research to identify the mechanisms, improve assessment, and develop targeted interventions is emerging and could open up new more comprehensive approaches to clinical care (Skidmore et al., 2015; Tay et al., 2021).

Finally, data science and artificial intelligence (AI) have grown exponentially in the healthcare space and more specifically in stroke rehabilitation (Ye et al., 2025; Liew et al., 2025; Zhang and Yu, 2025). While early AI applications focused primarily on diagnostics (e.g., image analysis for stroke detection), the field has expanded significantly into precision neurorehabilitation, including predictive modeling, adaptive treatment planning, and real-time therapy adjustment. However, clinical implementation of these precision rehabilitation approaches, especially for iterative and progressive interventions requiring repeated visits, remains limited. Emerging collaborative AI approaches in precision rehabilitation—in which AI systems work alongside clinicians and patients to co-create personalized treatment plans by integrating predictive models with clinical judgment, patient preferences, and real-time feedback—show promise but face substantial implementation challenges (Liew et al., 2025). These include accountability when AI recommendations lead to poor outcomes, data privacy concerns given the vast amounts of sensitive data required, integration complexity into clinical workflows, and clinician acceptance even when AI demonstrates efficacy. In contrast to standard RCTs, which must balance broad inclusion criteria to achieve adequate sample sizes against nosographic precision (e.g., “ischemic stroke” being too broad for mechanistic inference, yet narrower criteria reduce *N*), collaborative AI models explicitly leverage between-subject variability, using large, diverse datasets to generate individualized predictions and treatment recommendations that account for heterogeneity rather than controlling for it. This is an emergent and interdisciplinary area of rapidly moving research where mixed methods approaches are critical for ethical integration into health systems. While AI excels at processing large quantitative datasets to identify patterns and predict outcomes, qualitative methods are essential for: understanding patient and clinician perspectives on AI acceptability and trust; identifying barriers to implementation; capturing the nuanced, contextual factors that quantitative models may miss (such as motivation, fatigue, social support); and ensuring AI systems align with the values and preferences of those who use them. We believe collaborative AI holds transformative promise for precision neurorehabilitation, but realizing this potential will require deliberately integrating qualitative methods to ensure these systems are trustworthy, acceptable to patients and clinicians, and responsive to the complex, contextual realities of rehabilitation practice.

## 5 Conclusion

The future of stroke neurorehabilitation demands more than better technology and larger trials—it demands better questions. For too long, our field has prioritized what is easily measured over what is deeply meaningful to stroke survivors. While this approach has yielded important advances in motor rehabilitation, it has left critical gaps in our understanding of cognitive, perceptual, and affective/emotional recovery, and threatens to undermine emerging innovations in decision-making, sleep optimization, brain-computer interfaces, and AI-driven precision medicine. Integrating qualitative methods is not merely additive—it is transformative. It shifts our focus from what is conveniently quantified to what matters to patients, from intervention efficacy in controlled trials to effectiveness in lived contexts, from population averages to individual trajectories of recovery. Mixed methods approaches enable us to build theory grounded in lived experience, identify contextual factors that determine real-world effectiveness, reveal mechanisms that quantitative data alone cannot capture, and transform stroke survivors from study subjects into partners in knowledge creation. The evidence base we have built provides a strong foundation, but standing still means falling behind. Person-centered neurorehabilitation is not just better ethics; it is better science. What remains is the courage to embrace a more complete approach—one that honors both the precision of quantitative measurement and the richness of human experience. The field is ready. The time to act is now.

## Data Availability

The original contributions presented in the study are included in the article/supplementary material, further inquiries can be directed to the corresponding author.

## Footnotes

1 These investigators asked how patient-reported outcome which capture patient’s perspectives on the consequences of health and disease relate to performance-rated impairments. They found that performance-based and patient-reported measures formed distinct factors, consistent across recovery phases. Their findings reveal important differences between performance-rated and patient-reported outcomes, each with specific associated factors and neuroanatomy post-stroke. Incorporating sociodemographic and neuroanatomic characteristics into neurorehabilitation strategies may inform and optimize patient outcomes.
